# Nationwide Validation of the 8th American Joint Committee on Cancer TNM Staging System and Five Proposed Modifications for Resected Pancreatic Cancer

**DOI:** 10.1245/s10434-022-11664-4

**Published:** 2022-04-25

**Authors:** Thijs J. Schouten, Lois A. Daamen, Galina Dorland, Stijn R. van Roessel, Vincent P. Groot, Marc G. Besselink, Bert A. Bonsing, Koop Bosscha, Lodewijk A. A. Brosens, Olivier R. Busch, Ronald M. van Dam, Arantza Fariña Sarasqueta, Sebastiaan Festen, Bas Groot Koerkamp, Erwin van der Harst, Ignace H. J. T. de Hingh, Martijn Intven, Geert Kazemier, Vincent E. de Meijer, Vincent B. Nieuwenhuijs, G. Mihaela Raicu, Daphne Roos, Jennifer M. J. Schreinemakers, Martijn W. J. Stommel, M. F. van Velthuysen, Robert C. Verdonk, Joanne Verheij, Helena M. Verkooijen, Hjalmar C. van Santvoort, I. Quintus Molenaar, The Dutch Pancreatic Cancer Group

**Affiliations:** 1 Department of Surgery, Regional Academic Cancer Center Utrecht, UMC Utrecht Cancer Center & St. Antonius Hospital Nieuwegein, Utrecht, The Netherlands; 2 Department of Radiation Oncology, UMC Utrecht Cancer Center, Utrecht, The Netherlands; 3 Department of Surgery, Cancer Center Amsterdam, Amsterdam UMC, University of Amsterdam, Amsterdam, The Netherlands; 4 Department of Surgery, Leiden University Medical Center, Leiden, The Netherlands; 5 Department of Surgery, Jeroen Bosch Hospital, Den Bosch, The Netherlands; 6 Department of Pathology, UMC Utrecht Cancer Center, Utrecht, The Netherlands; 7 Department of Surgery, Maastricht UMC+, Maastricht, The Netherlands; 8 GROW - School for Oncology & Developmental Biology, Maastricht University, Maastricht, The Netherlands; 9 Department of General and Visceral Surgery, University Hospital Aachen, Aachen, Germany; 10 Department of Pathology, Leiden University Medical Center, Leiden, The Netherlands; 11 Department of Pathology, Cancer Center Amsterdam, Amsterdam UMC, University of Amsterdam, Amsterdam, The Netherlands; 12 Department of Surgery, OLVG, Amsterdam, The Netherlands; 13 Department of Surgery, Erasmus MC, Rotterdam, The Netherlands; 14 Department of Surgery, Maasstad Hospital, Rotterdam, The Netherlands; 15 Department of Surgery, Catharina Hospital, Eindhoven, The Netherlands; 16 Department of Surgery, Cancer Center Amsterdam, Amsterdam UMC, VU Medical Center, Amsterdam, The Netherlands; 17 Department of Surgery, University Medical Center Groningen, Groningen, The Netherlands; 18 Department of Surgery, Isala, Zwolle, The Netherlands; 19 Department of Pathology, St. Antonius Hospital, Nieuwegein, The Netherlands; 20 Department of Surgery, Reinier de Graaf Group, Delft, The Netherlands; 21 Department of Surgery, Amphia Hospital, Breda, The Netherlands; 22 Department of Surgery, Radboud University Medical Center, Nijmegen, The Netherlands; 23 Department of Pathology, Erasmus MC, Rotterdam, Netherlands; 24 Department of Gastroenterology, Regional Academic Cancer Center Utrecht, UMC Utrecht Cancer Center & St. Antonius Hospital Nieuwegein, Utrecht, The Netherlands; 25 Imaging Division, University Medical Center Utrecht, Utrecht, The Netherlands; 26 Utrecht University, Utrecht, The Netherlands

## Abstract

**Background:**

The prognostic value of four proposed modifications to the 8th American Joint Committee on Cancer (AJCC) TNM staging system has yet to be evaluated. This study aimed to validate five proposed modifications.

**Methods:**

Patients who underwent pancreatic ductal adenocarcinoma resection (2014–2016), as registered in the prospective Dutch Pancreatic Cancer Audit, were included. Stratification and prognostication of TNM staging systems were assessed using Kaplan–Meier curves, Cox proportional hazard analyses, and C-indices. A new modification was composed based on overall survival (OS).

**Results:**

Overall, 750 patients with a median OS of 18 months (interquartile range 10–32) were included. The 8th edition had an increased discriminative ability compared with the 7th edition {C-index 0.59 (95% confidence interval [CI] 0.56–0.61) vs. 0.56 (95% CI 0.54–0.58)}. Although the 8th edition showed a stepwise decrease in OS with increasing stage, no differences could be demonstrated between all substages; stage IIA vs. IB (hazard ratio [HR] 1.30, 95% CI 0.80–2.09; *p * = 0.29) and stage IIB vs. IIA (HR 1.17, 95% CI 0.75–1.83; *p * = 0.48). The four modifications showed comparable prognostic accuracy (C-index 0.59–0.60); however, OS did not differ between all modified TNM stages (ns). The new modification, migrating T3N1 patients to stage III, showed a C-index of 0.59, but did detect significant survival differences between all TNM stages (*p * < 0.05).

**Conclusions:**

The 8th TNM staging system still lacks prognostic value for some categories of patients, which was not clearly improved by four previously proposed modifications. The modification suggested in this study allows for better prognostication in patients with all stages of disease.

**Supplementary Information:**

The online version contains supplementary material available at 10.1245/s10434-022-11664-4.

Although recent advancements in pancreatic cancer treatment have led to more effective systemic therapy, pancreatic ductal adenocarcinoma (PDAC) remains associated with a 5-year survival of about 10%.^1^ For patients with resectable, non-metastasized disease, pancreatic resection combined with (neo)adjuvant systemic therapy is considered the most optimal treatment strategy.^2,3^ However, even after resection and (neo)adjuvant chemotherapy, oncological outcomes remain unsatisfactory, with a median overall survival (OS) of only 22 months.^3^

The prognosis of PDAC strongly depends on various pathological factors of the surgical specimen, including tumor size and metastatic lymph nodes, as well as any distant metastases.^4–9^ Consequently, staging after surgery is important for accurate survival predictions, to guide the direction of treatment strategies and to inform patients on their prognosis.^10,11^ To describe the extent of disease progression in patients with different types of cancer, the American Joint Committee on Cancer (AJCC) TNM staging system is commonly used.^12,13^ In 2018, the 8th edition of the AJCC TNM classification for PDAC was introduced, which resulted in adjustments regarding primary tumor (T) and regional lymph node (N) stage. The *T3* stage, previously defined as ‘tumor extension beyond the pancreas’, was changed to ‘tumors > 4 cm’, as the former definition could be interpreted differently by pathologists and lacked prognostic correlation.^12–14^ The extent of nodal involvement was subdivided from *N1* ‘regional lymph node metastasis’ into *N1* ‘metastases in 1–3 regional lymph nodes’, and *N2* ‘metastases in ≥ 4 regional lymph nodes’.^12,13^

The 8th edition of the AJCC TNM classification has been previously validated in high-volume pancreatic centers^15–26^; however, the general applicability of the revised classification, in particular to low-volume centers, remains unclear. To this purpose, validation in a nationwide setting is desired. In addition, four recent studies have proposed further modifications of the 8th TNM classification, suggesting increased discriminative power.^20–26^ Furthermore, a subdivision of N stage based on metastatic lymph node ratio (LNR), accounting for the total number of examined lymph nodes, was suggested for a more accurate prediction of survival.^24,27^

This study aims to evaluate and further improve the prognostic value and general applicability of the 8th TNM staging system for PDAC and proposed modifications in a nationwide, unselected cohort of patients.

## Methods

### Study Design

A nationwide, multicenter observational cohort study was conducted in all 16 centers for pancreatic cancer surgery in The Netherlands. Included were all patients who underwent resection of histologically proven PDAC between 2014 and 2016, as registered in the nationwide, mandatory, prospective Dutch Pancreatic Cancer Audit (DPCA).^28,29^ Exclusion criteria were macroscopic irradical resection (R2 resection) and death within 90 days after resection. Furthermore, patients who received neoadjuvant treatment were excluded, considering that consensus on optimal pathology assessment after neoadjuvant chemo(radio)therapy is lacking and may thus influence TNM staging.^30^ Patients with T4 tumors were also excluded, as stage T4 indicates unresectable tumors according to the most recent AJCC definition due to arterial tumor involvement.^13,20^ Moreover, the majority of these patients were considered to have locally advanced disease and were therefore initially treated with neoadjuvant chemotherapy.^31^

### Data Collection

After approval from the Dutch Pancreatic Cancer Group Scientific Committee, prospective baseline and perioperative data were retrieved from the clinical audit.^32^ The Charlson Age-Comorbidity Index (CACI) was calculated using the MDCalc CCI calculator.^33^ No data on race or ethnicity of study participants were obtained, as these data are not available in the DPCA. Survival data were retrieved from the patients’ hospital record. In addition, histopathological reports were obtained to collect detailed pathology data retrospectively. This information was used to assess T, N, and TNM stages according to the 7th and 8th AJCC definitions, based on tumor size, tumor extension, and lymph node involvement.^12,13^ Tumor size comprised the maximal tumor diameter in centimeters as mentioned in the pathology evaluation report, preferably measured microscopically. Resections were considered margin-positive if tumor cells were present within 1 mm of each microscopically assessed margin, apart from the anterior margin.^34^ The LNR was calculated by dividing the number of positive lymph nodes by the total number of lymph nodes examined. In case of uncertainty, an expert pancreatic cancer pathologist was consulted.

Pathology data were furthermore used to validate modifications of the 8th AJCC edition, as recently proposed in the literature.^23–26^ These modifications changed the TNM stages by either combining parameters from the 7th and 8th AJCC staging systems, altering their respective substages, or adjusting the N stage based on LNR (Table 1). In addition, a new modification was composed from optimal regrouping of the TNM substages based on median OS in our cohort, maintaining T and N stage definitions according to the 8th AJCC edition.^13^

**Table 1:** Definitions of the 7th and 8th editions of the AJCC TNM staging systems for PDAC, and proposed modifications of the 8th edition

7th AJCC staging system		8th AJCC staging system	
T1	Tumor limited to the pancreas, ≤ 2 cm in greatest dimension	T1	Maximum tumor diameter ≤ 2 cm
T2	Tumor limited to the pancreas, > 2 cm in greatest dimension	T2	Maximum tumor diameter > 2 cm and ≤ 4 cm
T3	Tumor extends beyond the pancreas but without involvement of the celiac axis or the superior mesenteric artery	T3	Maximum tumor diameter > 4 cm
T4	Tumor involves the celiac axis or the superior mesenteric artery (unresectable primary tumor)	T4	Tumor involves the celiac axis, the superior mesenteric artery, and/or common hepatic artery
N0	No regional lymph node metastasis	N0	No regional lymph node metastasis
N1	Regional lymph node metastasis	N1	Metastasis in 1–3 regional lymph nodes
		N2	Metastasis in ≥ 4 regional lymph nodes

7th AJCC edition				8th AJCC edition				Jiang et al. (2017) ^23^				Li et al. (2018) ^24^, ^a^				Shi et al. (2019) ^25^				Pu et al. (2019) ^26^				New modification^b^			
IA	T1	N0	M0	IA	T1	N0	M0	IA	T1	N0	M0	IA	T1	N0	M0	IA	T1	N0	M0	IA	T1	N0	M0	IA	T1	N0	M0
IB	T2	N0	M0	IB	T2	N0	M0	IB	T2-3	N0	M0	IB	T2	N0	M0	IB	T2	N0	M0	IB	T2	N0	M0	IB	T2	N0	M0
									T1 + ext.	N0	M0						T1	N1	M0								
IIA	T3	N0	M0	IIA	T3	N0	M0	IIA	T2 + ext.	N0	M0	IIA	T3	N0	M0	IIA	T3	N0	M0	II	T3	N0	M0	II	T3	N0	M0
									T1	N1-2	M0						T2	N1	M0		T1-3	N1	M0		T1	N1	M0
																	T1	N2	M0						T2	N1	M0
IIB	T1-3	N1	M0	IIB	T1-3	N1	M0	IIB	T3 + ext.	N0	M0	IIB	T1-3	N1	M0	IIB	T3	N1	M0					III	T1	N2	M0
									T2-3	N1	M0						T2	N2	M0								
III	T4	Nx	M0	III	T1-3	N2	M0	III	T2-3	N2	M0	III	T1-3	N2	M0	IIIA	T3	N2	M0	IIIA	T1-2	N2	M0		T3	N1	M0
					T4	Nx	M0									IIIB	T4	Nx	M0	IIIB	T3	N2	M0		T2	N2	M0
																					T4	Mx	M0		T3	N2	M0
IV	Tx	Nx	M1	IV	Tx	Nx	M1									IV	Tx	Nx	M1	IV	Tx	Nx	M1				

*AJCC* American joint committee on cancer, *T* Primary tumor, *N* Lymph nodes, *M* Distant metastasis, *ext.* Extension beyond the pancreas, *PDAC* Pancreatic ductal adenocarcinoma, *LNR* Lymph node ratio

^a^N stages are based on LNR: N0 (LNR = 0), N1 (LNR 0–0.45), N2 (LNR ≥0.45)

^b^The new modification was composed from optimal regrouping of the TNM substages based on median overall survival in our cohort, maintaining T and N stage definitions according to the 8th AJCC edition

### Statistical Analysis

Missing data were considered missing at random and handled by multiple imputation based on a Markov chain Monte Carlo method (5 imputations, 10 iterations).^35^ Categorical variables were presented as frequencies and percentages and were compared using the Chi-square test. Parametric continuous variables were presented as mean ± standard deviation (SD) and non-parametric continuous variables were presented as median (interquartile range [IQR]).

The primary outcome was OS, defined as the time from the date of primary tumor resection until the date of death from any cause. If survival data were missing, patients were censored from the date of their last follow-up. Survival rates after 1, 2, and 3 years were determined by patients with a known vital status at that respective time. Unadjusted OS was compared between and within the different T, N, and TNM stages of (proposed) staging systems using the Kaplan–Meier method and log-rank test, and presented as median with 95% confidence intervals (CIs). To assess the independent discriminative ability of the 8th T stage, Kaplan–Meier analysis was performed in node-negative patients only. Cox proportional hazard analyses were performed to obtain hazard ratios (HRs) with 95% CIs. To evaluate the discriminative power of the 7th and 8th TNM staging systems and their proposed modifications, concordance indices (C-index) were calculated. Statistical analyses were performed using R version 3.5.1 (Bell Laboratories, NH, USA), including the ‘survival’, ‘ggplot’ and ‘mice’ packages. A *p* value <0.05 was considered statistically significant.

## Results

### Study Population

A total of 750 patients who underwent PDAC resection were included, with a median follow-up time of 37 months (IQR 31–48 months) (Table 2). Mean age was 67 years (SD ± 9) and 402 patients (54%) were male. Mean pathological tumor size was 3.2 cm (SD ± 1.2). Median number of positive lymph nodes was 2 (IQR 0–5) and median number of examined lymph nodes was 15 (IQR 10–21). In 361 patients (48%), fewer than 15 lymph nodes were examined. Median OS of the entire cohort was 18 months (IQR 10–32 months), with a survival rate of 70, 41, and 29% after 1, 2, and 3 years of follow-up, respectively.

**Table 2:** Baseline characteristics of 750 patients who underwent resection of PDAC in the original cohort and dataset after multiple imputation

	Original cohort	Missing values [*n* (%)]	After multiple imputation
Age, years [mean ± SD]	67 ± 9	0 (0)	67 ± 9
Male sex [*n* (%)]	402 (54)	0 (0)	402 (54)
BMI [mean ± SD]	25 ± 4	3 (0)	25 ± 4
Charlson Age-Comorbidity Index [*n* (%)]		0 (0)	
< 4	404 (54)		404 (54)
≥ 4	346 (46)		346 (46)
ECOG performance score at primary diagnosis [*n* (%)]		290 (39)	
0	197 (43)		295 (39)
1	203 (44)		307 (41)
2	46 (10)		112 (15)
3	13 (3)		34 (5)
4	1 (0)		2 (0)
Preoperative serum CA19-9 [median (IQR)]	152 (38–500)	246 (33)	125 (34–480)
Type of surgery [*n* (%)]		2 (0)	
Open	672 (90)		674 (90)
Laparoscopic	70 (9)		70 (9)
Robot-assisted	6 (1)		6 (1)
Operation procedure [*n* (%)]		0 (0)	
Pancreatoduodenectomy	614 (82)		614 (82)
Distal pancreatectomy	104 (14)		104 (14)
Total pancreatectomy	32 (4)		32 (4)
Location tumor [*n* (%)]		0 (0)	
Head	639 (85)		639 (85)
Body/tail	111 (15)		111 (15)
Venous resection [*n* (%)]	182 (24)	2 (0)	182 (24)
Tumor size, cm [mean ± SD]	3.2 ± 1.2	10 (1)	3.2 ± 1.2
Tumor differentiation [*n* (%)]		77 (10)	
Well	89 (13)		96 (13)
Moderate	370 (55)		415 (55)
Poor	214 (32)		239 (32)
Microscopic lymphovascular invasion [*n* (%)]	383 (69)	192 (26)	499 (67)
Microscopic perineural invasion [*n* (%)]	595 (90)	88 (12)	671 (89)
Positive lymph nodes [median (IQR)]	2 (0–5)	1 (0)	2 (0–5)
Total lymph nodes [median (IQR)]	15 (10–21)	10 (1)	15 (10–21)
Lymph node ratio [*n* (%)]		10 (1)	
≤ 0.2	440 (59)		444 (59)
> 0.2	300 (41)		306 (41)
Resection margin status [*n* (%)]		2 (0)	
R0 > 1.0 mm	350 (47)		351 (47)
R1 ≤ 1.0 mm	398 (53)		399 (53)
T stage 7th AJCC edition [*n* (%)]		0 (0)	
T1	22 (3)		22 (3)
T2	42 (6)		42 (6)
T3	686 (91)		686 (91)
T stage 8th AJCC edition [*n* (%)]		10 (1)	
T1	113 (15)		114 (15)
T2	490 (66)		497 (66)
T3	137 (19)		139 (19)
N stage 7th AJCC edition [*n* (%)]		1 (0)	
N0	202 (27)		202 (27)
N1	547 (73)		548 (73)
N stage 8th AJCC edition [*n* (%)]		1 (0)	
N0	202 (27)		203 (27)
N1	290 (39)		290 (39)
N2	257 (34)		257 (34)
Major postoperative complications [*n* (%)]	178 (24)	0 (0)	178 (24)
Hospital stay, days [median (IQR)]	11 (8–16)	0 (0)	11 (8–16)
Adjuvant chemotherapy [*n* (%)]	473 (66)	29 (4)	486 (65)
Vital status [*n* (%)]		0 (0)	
Dead	565 (75)		565 (75)
Alive	185 (25)		185 (25)
Overall survival, months [median (IQR)]^a^	18 (10–32)		18 (10–32)

*PDAC* Pancreatic ductal adenocarcinoma, *SD* Standard deviation, *BMI* Body mass index, *ECOG* Eastern cooperative oncology group, *AJCC* American joint committee on cancer, *CA19-9* Carbohydrate antigen 19-9, *IQR* Interquartile range

^a^Overall survival was measured from the date of primary resection until the date of death or last follow-up

### Distribution of Patients

**Fig. 1:**
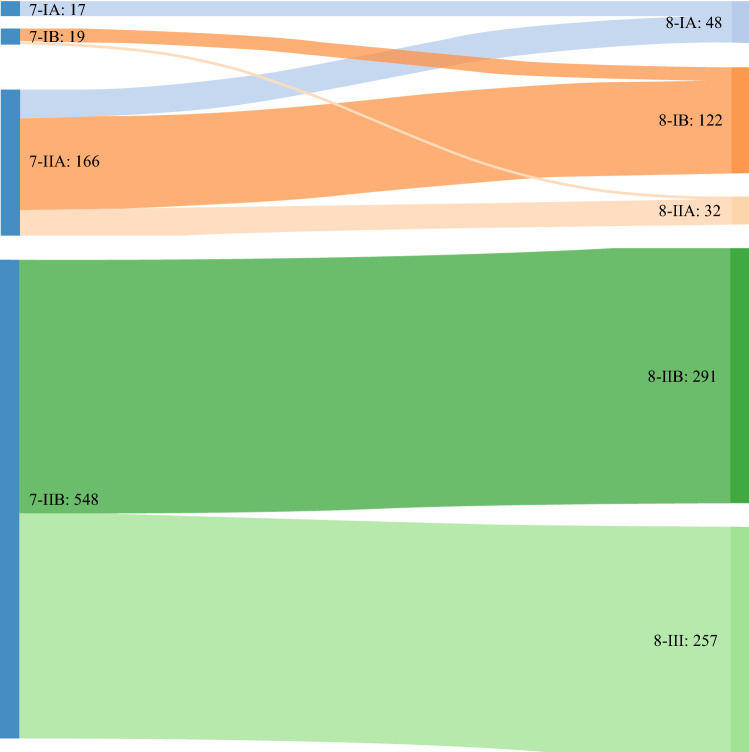
Sankey diagrams visualizing the reclassification of patients according to the various TNM staging systems. The colored blocks indicate different TNM stages for the 7th AJCC edition on the left and 8th AJCC edition on the right. *AJCC* American Joint Committee on Cancer

Regrouping of PDAC patients according to the 8th TNM classification, compared with the 7th edition, was visualized using a Sankey diagram (Fig. 1). The distribution of patients among the subgroups of the 7th TNM classification was considerably skewed. Restaging according to the 8th edition resulted in a reclassification of 394 patients (53%), of whom 137 patients (35%) migrated to a lower stage and 257 patients (65%) migrated to a higher stage. The revision mainly affected 7th TNM stage IIB; of 548 patients, 291 patients (53%) remained in stage IIB according to the 8th AJCC edition, while 257 patients (47%) were reclassified as stage III. This was mainly due to differentiation of N stage in the 8th edition. Consequently, the 8th TNM classification showed a more even distribution.

### Survival by T, N, and TNM Stages

**Fig. 2:**
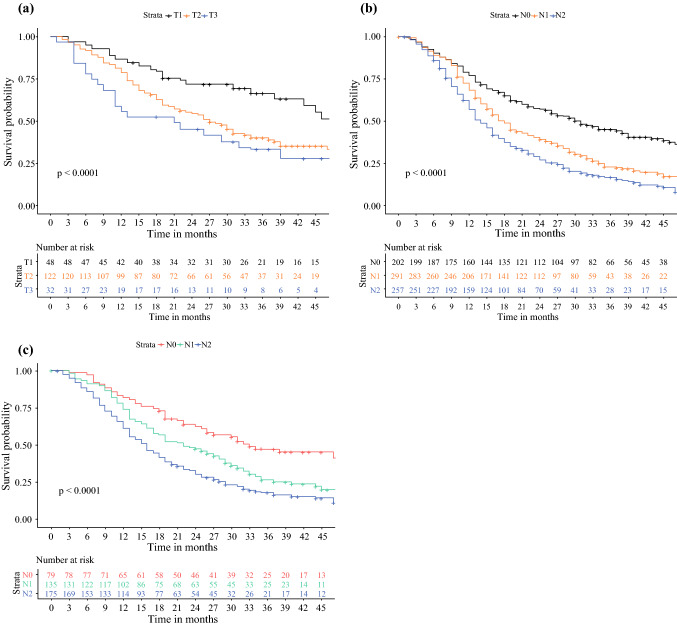
Kaplan–Meier curves comparing overall survival between each **a** T stage of the 8th AJCC edition in 202 patients with node-negative disease; **b** N stage of the 8th AJCC edition in all 750 patients; and **c** N stage of the 8th AJCC edition in 389 patients with at least 15 examined lymph nodes. *AJCC* American Joint Committee on Cancer

**Fig. 3:**
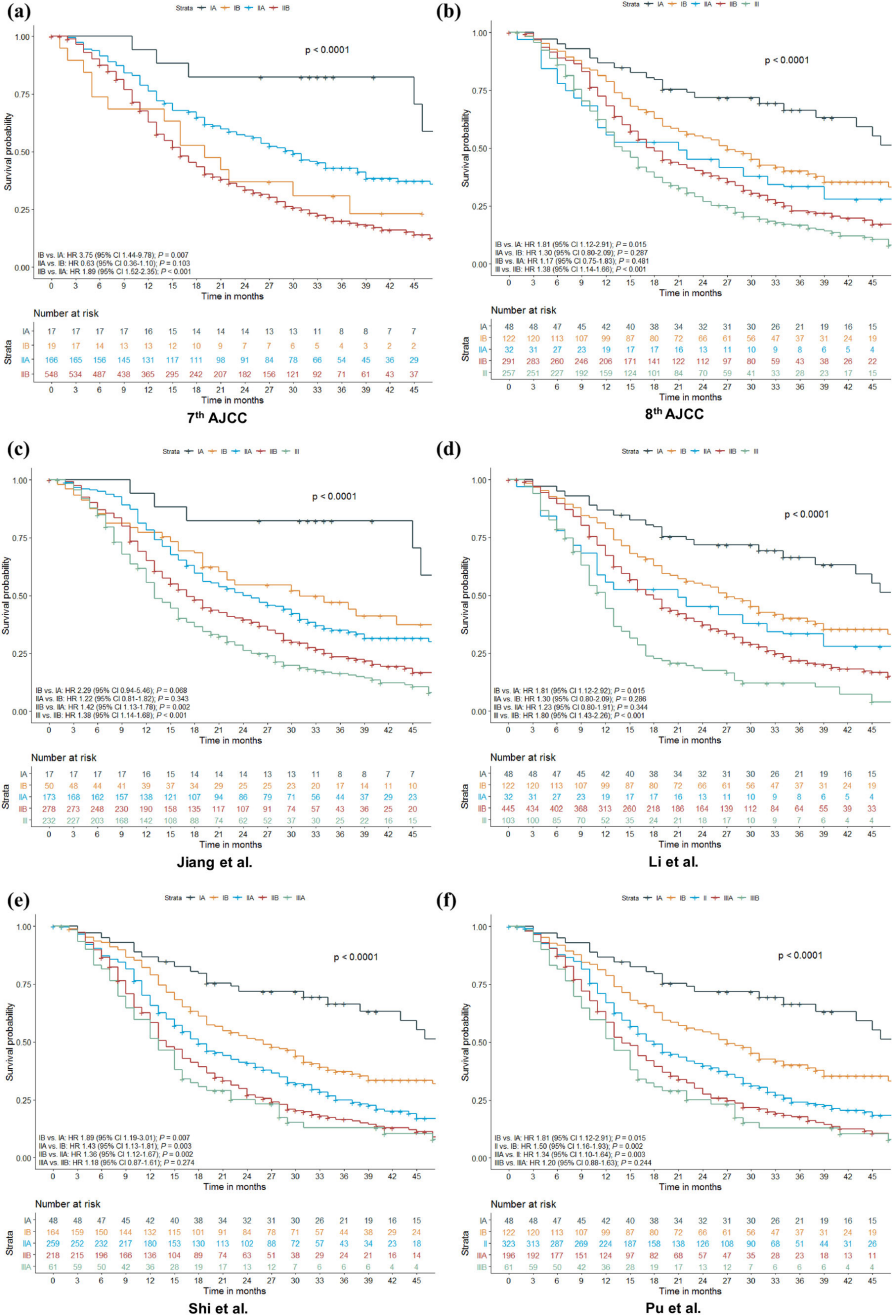
Kaplan–Meier curves and results of Cox proportional hazard analyses comparing overall survival between patients classified according to the **a** 7th AJCC TNM classification; **b** 8th AJCC TNM classification; **c** modification of Jiang et al. ^23^; **d** modification of Li et al. ^24^; **e** modification of Shi et al. ^25^; and **f** modification of Pu et al. ^26^ HRs are calculated between each consecutive stage. *AJCC* American Joint Committee on Cancer, *HR* hazard ratio, *CI* confidence interval

Both T and N stage of the 8th AJCC edition were discriminative for survival (Fig. 2a, b). A sensitivity analysis was performed, only using patients with at least 15 examined lymph nodes, which showed similar discrimination in survival for N stage (Fig. 2c). Kaplan–Meier and Cox proportional hazard analyses showed that a stepwise increase in TNM stage according to the 7th AJCC edition did not correspond with stepwise decrease in median OS (Fig. 3a). Stage IIA tumors were associated with a better OS than stage IB tumors, although this was not statistically significant (HR 0.63, 95% CI 0.36–1.10; *p* = 0.10). The 8th AJCC edition did show a sequential decline in OS from stage IA to III. However, no differences were found between stages IIA and IB (HR 1.30, 95% CI 0.80–2.09; *p * = 0.29) and stages IIB and IIA (HR 1.17, 95% CI 0.75–1.83; *p* = 0.48) (Fig. 3b).

### Proposed Modifications of the 8th Edition

Overall, the modifications of Jiang et al.^23^, Li et al.^24^ and Pu et al.^26^ did not clearly improve the distribution of patients among the subgroups compared with the 8th AJCC edition within this nationwide cohort (electronic supplementary material [ESM] Appendices IA, B, and D); however, the modification of Shi et al.^25^ did show a slight improvement (ESM Appendix IC).

Comparable with the 8th edition, all four proposed modifications showed an overall stepwise decrease in OS with increasing stage (*p *< 0.001). However, according to the modification of Jiang et al.^23^ no statistically significant survival differences were found between stages IB and IA (HR 2.29, 95% CI 0.94–5.46; *p* = 0.07) and stages IIA and IB (HR 1.22, 95% CI 0.81–1.82; *p *= 0.34) (Fig. 3c). With the modification of Li et al.^24^ OS was not significantly different between stages IIA and IB (HR 1.30, (95% CI 0.80–2.09; *p *= 0.29) and between stages IIB and IIA (HR 1.23, 95% CI 0.80–1.91; *p *= 0.34) (Fig. 3d). Moreover, no survival differences were found between stages IIIA and IIB according to the modification of Shi et al.^25^ (HR 1.18, 95% CI 0.87–1.61; *p* = 0.27) (Fig. 3e). Using the modification of Pu et al.^26^ OS did not significantly differ for stages IIIB and IIIA (HR 1.20, 95% CI 0.88–1.63; *p* = 0.24) (Fig. 3f).

### Prognostic Accuracy

The calculated C-indices for the 7th and 8th AJCC editions were 0.56 (95% CI 0.54–0.58) and 0.59 (95% CI 0.56–0.61), respectively (ESM Appendix II). From the proposed modifications, the modification by Shi et al.^25^ had the highest C-index of 0.60 (95% CI 0.57–0.62), compared with a C-index of 0.59 (95% CI 0.57–0.62) for Jiang et al.^23^, Li et al.^24^ and Pu et al.^26^

### New Modification

**Fig. 4:**
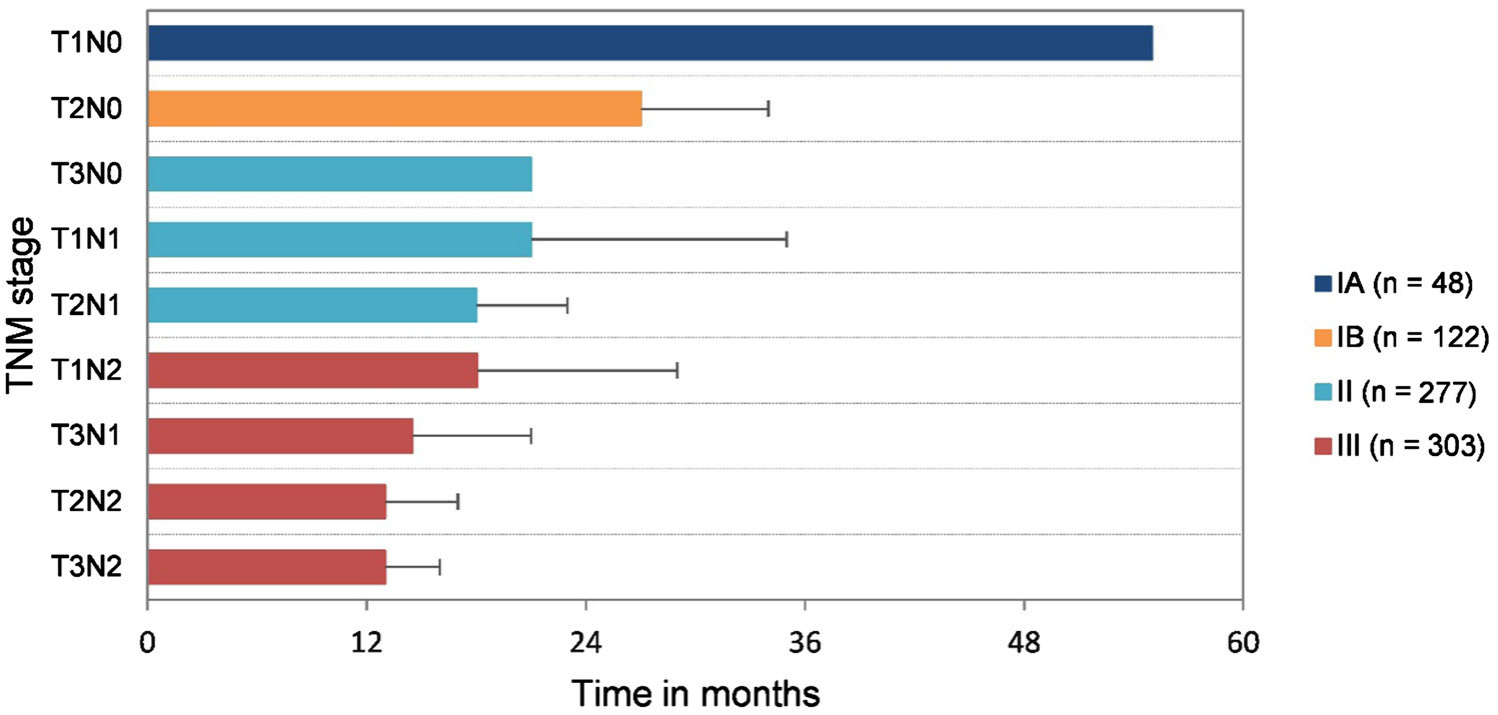
Median overall survival within the TNM (sub)stages according to our new modification.

**Fig. 5:**
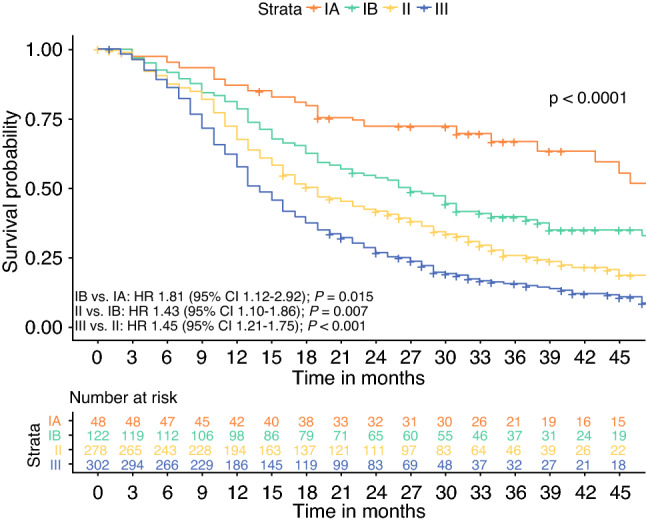
Kaplan–Meier curve and results of Cox proportional hazard analyses comparing overall survival in patients classified according to our new modification. Hazard ratios are calculated between each consecutive stage. *HR* hazard ratio, *CI* confidence interval

Based on median OS within each TNM substage, optimal regrouping of patients was performed to compose a new modification, maintaining the 8th AJCC T and N definitions (Fig. 4, Table 1). Utilizing this modification, patients with T3N1 disease migrate from stage II to stage III. This classification resulted in a more even distribution of patients and showed a significant decrease in OS between all subsequent TNM stages (*p *< 0.015) (Fig. 5). The C-index was 0.59 (95% CI 0.57–0.62) (ESM Appendix II).

## Discussion

This study validates the improved prognostic value and general applicability of the 8th AJCC TNM staging system after PDAC resection, compared with the 7th edition, in a nationwide, unselected cohort of patients. However, for patients with stage IIA disease, the prognostic value of the 8th TNM classification could still be questioned. In our cohort, the four proposed modifications of the 8th edition showed negligible improvement. Therefore, a new modification of the 8th AJCC edition was composed based on median OS of patients in our cohort, which resulted in optimal regrouping of the TNM substages.

The AJCC TNM staging system for malignant tumors is considered to be one of the most comprehensive tools for prognostication in patients with cancer in general.^36,37^ It allows investigators and doctors to communicate globally using a standardized language that reflects tumor burden. However, for PDAC patients, the TNM classification remains to be of moderate value.^12,38^ Accurate prediction of survival is important in cancer care as it helps to guide the direction of treatment decisions.^10^ Furthermore, the ability to correctly inform patients on their prognosis is crucial, in particular since the patients’ autonomy and shared decision making are increasingly being emphasized in current healthcare.^11^ Considering that recent advancements have resulted in more potent adjuvant treatment options for PDAC, such as FOLFIRINOX chemotherapy, accurate stratification of patients after PDAC resection might become of increasing importance.^2^

In recent years, the 8th AJCC TNM classification for PDAC was introduced, which showed major adjustments regarding the definitions of T and N stage.^13^ Using the former 7th edition, the majority of patients in our cohort was classified as stage IIA or IIB. This was mainly due to the large number of patients with T3 tumors, defined as tumors with ‘extension beyond the pancreas’.^12^ In contrast, with the 8th edition, a more even distribution among all TNM stages was found in our cohort, thus providing a better stratification for OS. Furthermore, the 8th AJCC classification showed a stage-dependent decrease in median OS, which was in contrast to its former edition.

However, with the 8th AJCC edition, OS remained indistinguishable between patients with stage IIA (including T3N0 patients) and stage IIB (including T1-3N1 patients) disease. This was in line with the findings of several other validation studies and may be explained by the relatively small number of T3N0 patients.^15,22,25,26^ Therefore, further improvement of the 8th TNM classification is mandatory. To this purpose, various modifications of the 8th edition were proposed, which showed only minor improvements within our cohort.^19,23–26^ Of these modifications, the regrouping scheme of Shi et al.^25^ using unchanged TNM parameters (Table 1) demonstrated the highest prognostic value.^25^ However, with their modification, OS could not be distinguished between patients with stage IIB and IIIA disease. Although our new modification, regrouping substages within stages II and III, showed a comparable discriminative power, significant OS differences between each revised subgroup were shown. Therefore, the newly proposed modification allows for better prognostication in patients with all stages of disease after PDAC resection.

Furthermore, various studies reported conflicting results with regard to the independent prognostic value of T and N stage. A dual-center study found that the revised T stage improves prognostication, while the revised N stage did not.^39^ On the contrary, a multicenter study by van Roessel et al. did not find a correlation between OS and the revised T stage in node-negative patients, whereas the revised N stage provided accurate discrimination of OS.^19^ Nevertheless, most studies proposing modifications of the 8th TNM classification maintained the 8th AJCC definitions for T and N stage.^25,26,40,41^ In our cohort, the 8th T and N stages were both found to be associated with OS, thereby significantly adding to the prognostic ability of the TNM staging system.

The present study has some limitations. First, due to lack of standardization, not all centers used the same histopathological examination and reporting protocol. Nevertheless, the nationwide pathology registry and network (PALGA) released a pathology protocol for synoptic reporting of pancreatic cancer in 2015.^42^ As all pathology laboratories in The Netherlands are affiliated with this network, standardization was stimulated. Second, patients who received neoadjuvant treatment were excluded. Neoadjuvant chemo(radio)therapy induces reactive changes to the pancreatic specimen such as fibrosis, which complicates the macroscopic and microscopic assessment and measurement of the tumor.^30,43^ Consequently, the current results are not applicable to patients who were treated with neoadjuvant therapy. Although international consensus was recently reached on assessment of the pancreatic specimen after neoadjuvant therapy, future research should validate the TNM classification in this setting.^44^ Third, to accurately assess the N stage, and thus the overall TNM stage, a sufficient number of harvested lymph nodes is mandatory.^45,46^ A higher number of harvested lymph nodes decreases the likelihood of underestimating the N stage. According to the International Study Group of Pancreatic Surgery (IGSPS), it is recommended to harvest at least 15 lymph nodes during PDAC resection.^47^ According to this criterion, the total number of examined lymph nodes was insufficient for a substantial part of patients (48%) in our cohort. It might therefore be possible that the number of positive lymph nodes in our study population, and subsequently the true N1 and N2 rates, are actually higher than reported, although a sensitivity analysis only using patients with sufficient harvested lymph nodes showed similar discrimination for N stage. This could be a consequence of the nationwide design of the study, which may have led to heterogeneity in the extent of lymph node harvesting. Despite that this could have influenced our results, it reflects the TNM assessment in a ‘real world’ daily clinical practice and therefore increases the general applicability of our findings*.*

## Conclusion

This study provides evidence on the importance of joint consideration of T and N stage, and helps to further improve the TNM classification for PDAC. Although the prognostic value and general applicability of the 8th AJCC TNM staging system is improved compared with its former 7th edition, it still lacks prognostic value for some categories of patients. We propose a modification that moves patients with T3N1 disease from stage II to stage III. This revision allows for a better stepwise prognostication, although external validation is required to determine its true prognostic value.

## Supplementary Information

Below is the link to the electronic supplementary material.

Supplementary file1 (PDF 221 kb)
