# Correction to: Nationwide Validation of the 8th American Joint Committee on Cancer TNM Staging System and Five Proposed Modifications for Resected Pancreatic Cancer

**DOI:** 10.1245/s10434-022-12182-z

**Published:** 2022-07-07

**Authors:** Thijs J. Schouten, Lois A. Daamen, Galina Dorland, Stijn R. van Roessel, Vincent P. Groot, Marc G. Besselink, Bert A. Bonsing, Koop Bosscha, Lodewijk A. A. Brosens, Olivier R. Busch, Ronald M. van Dam, Arantza Fariña Sarasqueta, Sebastiaan Festen, Bas Groot Koerkamp, Erwin van der Harst, Ignace H. J. T. de Hingh, Martijn Intven, Geert Kazemier, Vincent E. de Meijer, Vincent B. Nieuwenhuijs, G. Mihaela Raicu, Daphne Roos, Jennifer M. J. Schreinemakers, Martijn W. J. Stommel, M. F. van Velthuysen, Robert C. Verdonk, Joanne Verheij, Helena M. Verkooijen, Hjalmar C. van Santvoort, I. Quintus Molenaar, The Dutch Pancreatic Cancer Group

**Affiliations:** 1 Department of Surgery, Regional Academic Cancer Center Utrecht, UMC Utrecht Cancer Center & St. Antonius Hospital Nieuwegein, Utrecht, The Netherlands; 2 Department of Radiation Oncology, UMC Utrecht Cancer Center, Utrecht, The Netherlands; 3 Department of Surgery, Cancer Center Amsterdam, Amsterdam UMC, University of Amsterdam, Amsterdam, The Netherlands; 4 Department of Surgery, Leiden University Medical Center, Leiden, The Netherlands; 5 Department of Surgery, Jeroen Bosch Hospital, Den Bosch, The Netherlands; 6 Department of Pathology, UMC Utrecht Cancer Center, Utrecht, The Netherlands; 7 Department of Surgery, Maastricht UMC+, Maastricht, The Netherlands; 8 GROW - School for Oncology & Developmental Biology, Maastricht University, Maastricht, The Netherlands; 9 Department of General and Visceral Surgery, University Hospital Aachen, Aachen, Germany; 10 Department of Pathology, Leiden University Medical Center, Leiden, The Netherlands; 11 Department of Pathology, Cancer Center Amsterdam, Amsterdam UMC, University of Amsterdam, Amsterdam, The Netherlands; 12 Department of Surgery, OLVG, Amsterdam, The Netherlands; 13 Department of Surgery, Erasmus MC, Rotterdam, The Netherlands; 14 Department of Surgery, Maasstad Hospital, Rotterdam, The Netherlands; 15 Department of Surgery, Catharina Hospital, Eindhoven, The Netherlands; 16 Department of Surgery, Cancer Center Amsterdam, Amsterdam UMC, VU Medical Center, Amsterdam, The Netherlands; 17 Department of Surgery, University Medical Center Groningen, Groningen, The Netherlands; 18 Department of Surgery, Isala, Zwolle, The Netherlands; 19 Department of Pathology, St. Antonius Hospital, Nieuwegein, The Netherlands; 20 Department of Surgery, Reinier de Graaf Group, Delft, The Netherlands; 21 Department of Surgery, Amphia Hospital, Breda, The Netherlands; 22 Department of Surgery, Radboud University Medical Center, Nijmegen, The Netherlands; 23 Department of Pathology, Erasmus MC, Rotterdam, Netherlands; 24 Department of Gastroenterology, Regional Academic Cancer Center Utrecht, UMC Utrecht Cancer Center & St. Antonius Hospital Nieuwegein, Utrecht, The Netherlands; 25 Imaging Division, University Medical Center Utrecht, Utrecht, The Netherlands; 26 Utrecht University, Utrecht, The Netherlands

## Correction to: Annals of Surgical Oncology 10.1245/s10434-022-11664-4

In the original online version of the article Fig. 4 and the last sentence in the Proposed Modifications of the 8th Edition section were incorrect.

The original article was corrected.

