# Supplementary material for: Nationwide Validation of the 8th American Joint Committee on Cancer TNM Staging System and Five Proposed Modifications for Resected Pancreatic Cancer
Source: Ann Surg Oncol. 2022 Apr 25;29(9):5988–99. doi: 10.1245/s10434-022-11664-4 (PMC9356941; doi:10.1245/s10434-022-11664-4)
Supplement: Supplementary file 1 — Supplementary file1 (PDF 221 kb) [file 10434_2022_11664_MOESM1_ESM.pdf]

Appendix I. Sankey diagrams visualizing the reclassification of patients according to the various TNM staging systems. The colored blocks indicate different TNM stages for the **(A)** 8th AJCC edition on the left and modification of Jiang et al. on the right; **(B)** 8th AJCC edition on the left and modification of Li et al. on the right; **(C)** 8th AJCC edition on the left and modification of Shi et al. on the right; and **(D)** 8th AJCC edition on the left and modification of Pu et al. on the right.

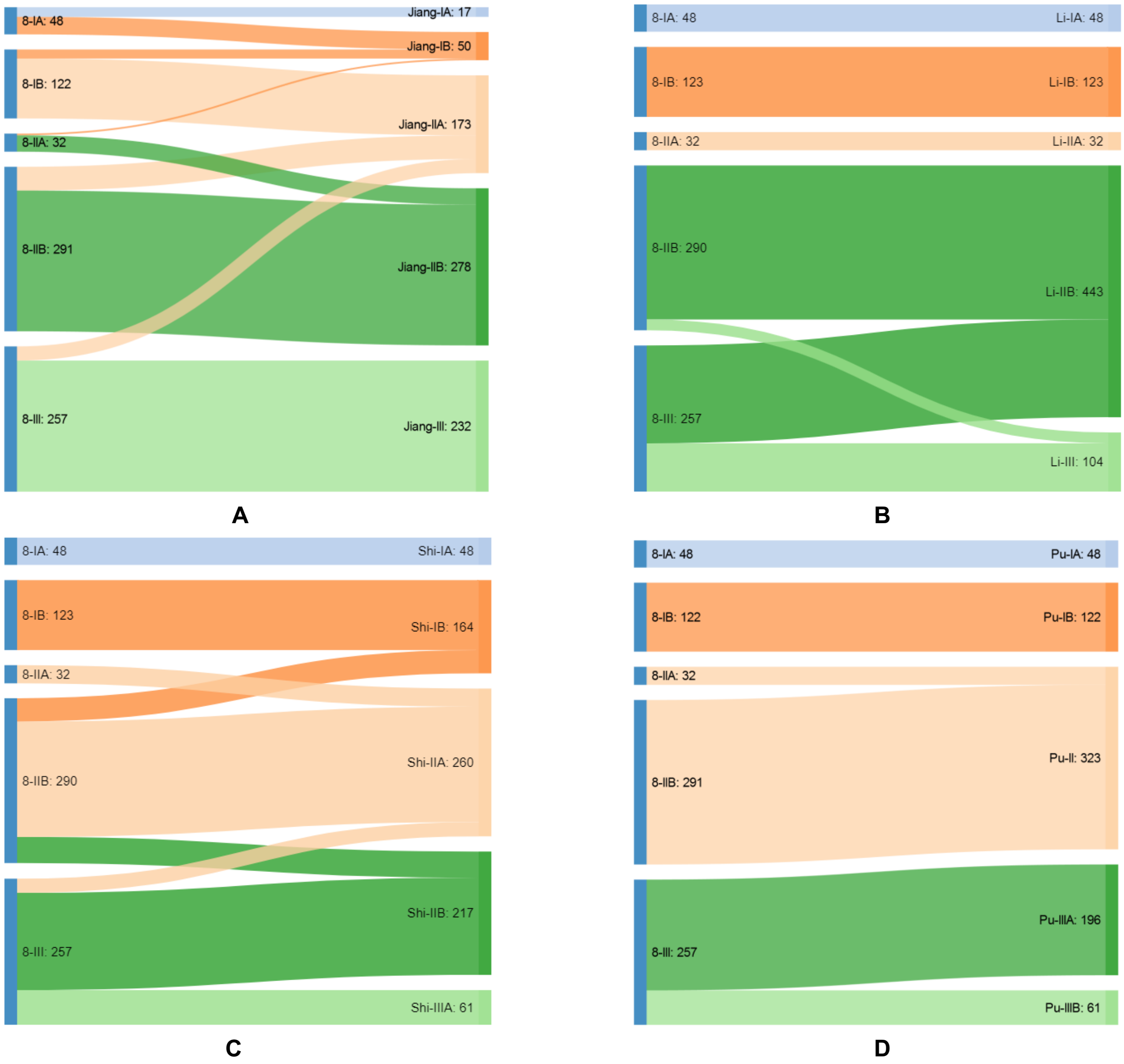

Appendix II. C-indices of different TNM staging systems

Staging system	C-index	95% CI
7th AJCC edition	0.56	0.54-0.58
8th AJCC edition	0.59	0.56-0.61
Jiang et al. (2017)	0.59	0.57-0.62
Li et al. (2018)	0.59	0.57-0.62
Shi et al. (2019)	0.60	0.57-0.62
Pu et al. (2019)	0.59	0.57-0.62
New modification	0.59	0.57-0.62

AJCC, American Joint Committee on Cancer; C-index, concordance index
